# Obesity and COVID-19 severity independently shape adipokine dysregulation and immune cell remodeling

**DOI:** 10.3389/fimmu.2026.1890251

**Published:** 2026-09-08

**Authors:** Karla Jimena Basilio-Aguilar, Alfredo Pérez-Fragoso, Edith Cerón-Cruz, Abdiel Absalón-Aguilar, Ingrid Itzayanna Ortega-Mejia, Brian Bernal-Alferes, Nayeli Romero-López, Juan Padierna-Olivos, Luis O. Soto-Rojas, Citlaltépetl Salinas-Lara, Adolfo René Méndez-Cruz, María Lilia Domínguez-López, Diana Gómez-Martín, Jiram Torres-Ruíz, José Pablo Romero-López

**Affiliations:** 1 Red Medicina para la Educación, el Desarrollo y la Investigación Científica de Iztacala (MEDICI), Carrera de Médico Cirujano, Facultad de Estudios Superiores Iztacala, Universidad Nacional Autónoma de México (UNAM), Tlalnepantla de Baz, Estado de México, Mexico; 2 Posgrado en Ciencias Quimicobiológicas, Escuela Nacional de Ciencias Biológicas, Instituto Politécnico Nacional (IPN), Ciudad de México, Mexico; 3 Departamento de Inmunología y Reumatología, Instituto Nacional de Ciencias Médicas y Nutrición Salvador Zubirán (INCMNSZ), Ciudad de México, Mexico; 4 Posgrado en Ciencias Biológicas, Universidad Nacional Autónoma de México, Estado de México, Mexico; 5 Laboratorios de Especialidades Inmunológicas, Ciudad de México, Mexico; 6 Dpto. de Neuropatología, Instituto Nacional de Neurología y Neurocirugía Manuel Velasco Suárez, Ciudad de México, Mexico

**Keywords:** adipokines, ART-ANOVA, CD8 Treg, COVID-19, disease severity, immune dysregulation, ISG15, low-density granulocytes

## Abstract

**Background:**

Obesity is a major risk factor for severe COVID-19, yet the immunological mechanisms by which it modifies disease progression remain incompletely characterized. We aimed to dissect the independent and interactive contributions of obesity and COVID-19 severity on circulating adipokines, cytokines, and immune cell populations.

**Methods:**

Sixty patients with confirmed SARS-CoV-2 infection were stratified by disease severity (Mild/Moderate, n = 8; Severe, n = 30; Critical, n = 22) and obesity status (persons without obesity [PwoO], n = 30; persons with obesity [PwO], n = 30; BMI threshold 30 kg/m²). Serum TNF-α, resistin, IL-1β, IL-8, and NETs-ISG15 complexes were measured alongside circulating Th1, Th2, Th17, CD8 Treg, CD8 Tcm, intermediate monocytes, and low-density granulocytes (LDGs). Statistical analysis used Aligned Rank Transform ANOVA (ART-ANOVA), followed by Kruskal–Wallis and Dunn’s *post-hoc* test with Holm adjustment within each obesity stratum.

**Results:**

ART-ANOVA identified three distinct immunological patterns. First, TNF-α and resistin were significantly elevated by obesity as independent main effects (p = 0.012 and p = 0.026), with resistin additionally driven by severity (p = 0.0001). Second, IL-1β, IL-8, and NETs-ISG15 complexes were governed by disease severity (p = 0.0003, p = 0.0003, p = 0.002); IL-1β showed a symmetric Severe-to-Critical elevation across obesity strata, whereas IL-8 and NETs-ISG15 were selectively amplified in PwO at the critical stage (Severe vs. Critical: IL-8 p = 0.0002; NETs-ISG15 p = 0.015). Third, CD8 Treg cells and LDGs showed the largest severity-driven effects (both p < 0.0001), with symmetric depletion and expansion across obesity strata, identifying them as severity biomarkers independent of metabolic background. Th17 cells were suppressed by obesity (p = 0.001) and severity (p = 0.021). CD8 Tcm exhibited a unique obesity × severity interaction (p = 0.009), with a paradoxical expansion in critical PwO patients, absent in PwoO.

**Conclusion:**

Obesity and COVID-19 severity exert distinct, non-interacting effects on the immune landscape, with three exceptions: TNF-α and IL-8/NETs-ISG15 show obesity-conditional amplification of severity, and CD8 Tcm undergoes an obesity-specific expansion at the critical stage. These findings provide a mechanistic framework for obesity-modified immune responses in COVID-19 and identify candidate biomarkers for severity stratification.

## 1 Introduction

The coronavirus disease 2019 (COVID-19) pandemic, caused by severe acute respiratory syndrome coronavirus 2 (SARS-CoV-2), has profoundly impacted global health systems since its emergence in late 2019. The clinical spectrum of COVID-19 ranges from asymptomatic or mild upper respiratory symptoms to severe pneumonia, acute respiratory distress syndrome (ARDS), and multi-organ failure (1). Severe COVID-19 is characterized by profound dysregulation of both innate and adaptive immune responses, including excessive cytokine production, lymphocyte dysfunction, aberrant myeloid activation, and impaired interferon signaling (2, 3). However, the extent to which these immunological alterations are intrinsically linked to disease severity versus shaped by pre-existing immunometabolic conditions remains incompletely understood.

Among the comorbidities associated with poor COVID-19 outcomes, obesity emerged as one of the most prevalent and clinically relevant risk factors worldwide. Individuals with obesity exhibit increased risk of hospitalization, invasive mechanical ventilation, and mortality following SARS-CoV-2 infection (4). Obesity is characterized by chronic low-grade inflammation and profound immune dysregulation, driven by adipose tissue remodeling and altered adipokine secretion, with increased production of pro-inflammatory mediators (5). This association is particularly concerning in countries with high obesity prevalence, such as Mexico, where approximately 36% of adults are classified as having obesity (6).

The mechanisms linking obesity to severe COVID-19 outcomes are multifactorial and incompletely understood. Mechanical factors, including reduced lung compliance and impaired ventilation due to excess adipose tissue, contribute to respiratory compromise in patients with obesity (7). Obesity-associated adipose tissue remodeling promotes sustained production of pro-inflammatory adipokines and cytokines that can alter systemic immune homeostasis. Elevated leptin and resistin levels, together with reduced adiponectin signaling, contribute to chronic activation of myeloid and lymphoid compartments and may predispose individuals with obesity to exaggerated inflammatory responses during viral infection (8).

In the context of obesity, adipose tissue undergoes pathological remodeling characterized by adipocyte hypertrophy, hypoxia, macrophage infiltration, and a shift toward pro-inflammatory adipokine secretion (9). Leptin, the prototypical adipokine, is elevated in obesity and promotes pro-inflammatory responses, including the activation of monocytes, macrophages, and T cells (10). Conversely, adiponectin (which possesses anti-inflammatory and insulin-sensitizing properties) is paradoxically reduced in obesity despite its adipose tissue origin (9). Resistin, another adipokine elevated in obesity, has been implicated in insulin resistance and inflammatory signaling (11). These adipokine imbalances may predispose individuals with obesity to exaggerated inflammatory responses upon SARS-CoV-2 infection.

Emerging evidence suggests that adipokines may directly influence COVID-19 pathogenesis and outcomes. Several studies have reported altered circulating levels of leptin, adiponectin, and resistin in patients with COVID-19 compared with healthy controls, and some have identified associations between specific adipokine profiles and disease severity (12). For example, elevated leptin levels have been associated with increased inflammatory markers and worse clinical outcomes in some cohorts (4), while the role of adiponectin remains controversial, with studies reporting both protective and detrimental associations depending on disease stage and patient characteristics (13, 14)Resistin has been positively correlated with inflammatory and coagulation markers in COVID-19 patients (15).

Beyond their direct effects on inflammation, adipokines modulate the function of diverse immune cell populations that are critical to antiviral responses and immunopathology in COVID-19. T lymphocytes, particularly CD4+ T helper (Th) subsets and CD8+ T cells, play essential roles in coordinating adaptive immune responses and eliminating virus-infected cells (1). Severe COVID-19 is associated with profound lymphopenia and functional exhaustion of T cells, particularly affecting CD4+ and CD8+ populations (16). Within CD4+ T cells, distinct functional subsets, including Th1 cells (producing interferon-γ), Th2 cells (producing interleukin-4), and Th17 cells (producing interleukin-17), contribute differentially to antiviral immunity and immunopathology. Regulatory T cells (Tregs), including CD8+ Treg populations, are critical for maintaining immune homeostasis and preventing excessive inflammation (2). Memory T cell subsets, such as central memory T cells (Tcm), are important for long-term immunity and recall responses.

Innate immune cells also play pivotal roles in COVID-19 pathogenesis. Monocytes, which can be classified into classical (CD14+CD16−), intermediate (CD14+CD16+), and non-classical (CD14lowCD16+) subsets based on surface marker expression, exhibit distinct functional properties and trafficking patterns (17). For example, intermediate monocytes have been implicated in inflammatory responses and tissue damage in severe COVID-19. On the other hand, low-density granulocytes (LDGs) (18). LDGs have also been linked to enhanced neutrophil extracellular trap (NET) formation, endothelial injury, and immunothrombosis in severe SARS-CoV-2 infection (19).

Cytokines and chemokines represent another critical layer of immune regulation in COVID-19. Pro-inflammatory cytokines such as tumor necrosis factor-α (TNF-α), interleukin-1β (IL-1β), and interleukin-8 (IL-8) are elevated in severe COVID-19 and contribute to the cytokine storm associated with ARDS and multi-organ failure (20). Interferon-stimulated gene 15 (ISG15), induced by type I interferons and involved in antiviral defense, can also be released extracellularly and, in inflammatory contexts, associates with neutrophil extracellular traps (NETs) as free (non-conjugated) ISG15, where it exerts cytokine-like pro-inflammatory activity. Dysregulated interferon responses and increased NET formation have both been documented in severe COVID-19, and their convergence in the form of circulating NETs-ISG15 complexes represents a potential mechanism of immune amplification in the critical stage of disease (21).

Despite growing recognition of the importance of adipokines and immune dysregulation in COVID-19, most research has been conducted in populations of European or Asian ancestry, with limited data from Latin American populations, such as those in Mexico, where genetic background, obesity prevalence, and healthcare contexts differ substantially (6, 19, 20).

In this study, we sought to disentangle the independent and interactive effects of obesity and COVID-19 severity on circulating adipokines, inflammatory mediators, and innate and adaptive immune cell populations in hospitalized patients with SARS-CoV-2 infection. Using a factorial non-parametric analytical approach, we identified distinct immunological patterns associated with obesity, disease severity, and selective obesity-conditioned amplification pathways, providing a framework for understanding immunometabolic remodeling in severe COVID-19.

## 2 Materials and methods

### 2.1 Study design and participants

This cross-sectional observational study enrolled 60 adults with confirmed SARS-CoV-2 infection (RT-PCR-positive) who were admitted to the Instituto Nacional de Ciencias Médicas y Nutrición Salvador Zubirán (INCMNSZ), Mexico City, Mexico, between March and August 2020. Patients with autoimmune disease, active immunosuppressive therapy, HIV infection, transplant recipients, active neoplastic disease, or other active systemic infections were excluded. All participants provided written informed consent prior to enrollment. The study was approved by the Institutional Ethics and Research Committees of INCMNSZ (reference: 3341) and was conducted in accordance with the Declaration of Helsinki.

Patients were classified into three severity groups at admission according to established criteria: (i) Mild/Moderate: RT-PCR-confirmed SARS-CoV-2 infection with no criteria for severe or critical disease; (ii) Severe: any of respiratory rate >30 breaths/min, peripheral oxygen saturation (SpO_2_) <93%, or PaO_2_/FiO_2_ ratio <300 mmHg; (iii) Critical: fulfilment of severe criteria plus requirement for invasive mechanical ventilation, septic shock, or evidence of multi-organ failure.

Obesity was defined as BMI ≥30 kg/m² (World Health Organization criteria), and participants were classified into persons without obesity (PwoO; BMI <30 kg/m², n = 30) and persons with obesity (PwO; BMI ≥30 kg/m², n = 30). This terminology is used throughout to avoid stigmatizing language (24).

Detailed pharmacological and supportive treatment data (e.g., corticosteroids, antiviral agents, anticoagulation regimens, and respiratory support modality) were not systematically recorded in the study database and could therefore not be analyzed as a covariate in this cohort.

### 2.2 Biological sample collection and laboratory measurements

Peripheral blood was collected at the time of clinical assessment and processed within 4 hours. Serum concentrations of TNF-α, IL-1β, IL-8 and resistin were simultaneously quantified using the LEGENDplex™ Human Adipokine Panel Standard 13-plex bead-based immunoassay (BioLegend^®^, San Diego, CA, USA), following the manufacturer’s protocol. Samples were acquired by flow cytometry and analyzed using Qnoit^®^ software (BioLegend^®^).

Plasma NETS-ISG15 complexes, indicative of ISG15 physically associated with circulating neutrophil extracellular traps (NETs), were quantified by an in-house capture ELISA as previously described (21). Briefly, high-binding 96-well plates were coated overnight at 4 °C with rabbit polyclonal anti-human ISG15 antibody at 1:100 dilution (Abgent, Cat. No. AP1150a) in coating buffer. After three washes with PBS/Tween 20, non-specific binding sites were blocked with 1% bovine serum albumin (BSA) in PBS for 6 h at room temperature. Plasma samples were diluted 1:10 in 1% BSA and incubated overnight at 4 °C. Following three additional washes with PBS/Tween 20, wells were incubated with anti-DNA peroxidase-conjugated antibody (clone MCA-33) supplied with the Cell Death Detection ELISA-PLUS kit (Roche, Cat. No. 11774425001, Basel, Switzerland). After five final washes with PBS/Tween 20, the reaction was developed with TMB substrate (Thermo Fisher Scientific, MA, USA), and absorbance was read at 450 nm after applying the stop solution. Results were expressed as an optic density index (ODI).

Peripheral blood mononuclear cells (PBMCs) were isolated from 20 mL of EDTA- anticoagulated venous blood by density gradient centrifugation using Ficoll-Paque PLUS (GE Healthcare™). Cells were stained with the Zombie Aqua™ viability marker (BioLegend^®^) and incubated with fluorochrome-conjugated monoclonal antibodies (all BioLegend^®^, San Diego, CA, USA) (**Supplementary Table 1**). For intracytoplasmic cytokine staining, PBMCs were stimulated with phorbol 12-myristate 13-acetate (PMA; 50 ng/mL) and ionomycin (1 μg/mL) in the presence of monensin for 5 hours at 37 °C, then fixed and permeabilized using BD Cytofix/Cytoperm™ Buffer. Cell populations were defined as follows: Th1 (CD3+CD4+IFN-γ+), Th2 (CD3+CD4+IL-4+), Th17 (CD3+CD4+IL-17A+), CD8 central memory T cells—Tcm—(CD3+CD8+CD45RO+CD62L+CCR7+), CD8 regulatory T cells—CD8 Treg—(CD3+CD8+CD25+CD127low), intermediate monocytes (CD14+CD16+), and low-density granulocytes (LDGs; CD14−CD10−CD15+), isolated from the PBMC fraction. Absolute counts (cells/μL) were calculated using leucocyte subset counts from a same-day complete blood count. Data were acquired on a BD LSR Fortessa 4-laser flow cytometer (BD Biosciences, New Jersey, USA), with a minimum of 1,000,000 events collected per sample.

### 2.3 Statistical analysis

Sample size calculations were not performed *a priori*; the cohort comprises all eligible patients recruited between March and August 2020. Group sizes were: PwoO-Mild/Moderate n = 6, PwoO-Severe n = 13, PwoO-Critical n = 11, PwO-Mild/Moderate n = 2, PwO-Severe n = 17, PwO-Critical n = 11 (total N = 60).

Continuous variables are reported as median (Q1–Q3). Normality was assessed using the Shapiro–Wilk test; all variables were non-normally distributed, warranting the use of non-parametric methods throughout. To partition variance between the two design factors and their interaction, we applied the Aligned Rank Transform ANOVA (ART-ANOVA) (22, 23); to each variable, with obesity (two levels: PwoO, PwO) and severity (three levels: Mild/Moderate, Severe, Critical) as fixed between-subjects factors. ART aligns the response variable separately for each effect, ranks the aligned values, and applies standard factorial ANOVA F-statistics to the rank-aligned series.

*Post-hoc* pairwise comparisons across severity levels were conducted within each obesity stratum using the Kruskal–Wallis test followed by Dunn’s test with Holm step-down adjustment. Effects were considered statistically significant at p < 0.05. Significance levels are denoted as * p < 0.05, ** p < 0.01, *** p < 0.001, **** p < 0.0001.

All analyses were performed in Python 3.12 using pandas, SciPy, scikit-posthocs, and statsmodels; ART-ANOVA was implemented following Higgins and Wobbrock et al. (22). Descriptive statistics and primary data organization were performed in GraphPad Prism v8.0.1 (GraphPad Software, San Diego, CA, USA).

## 3 Results

### 3.1 Study population and clinical characterization

Sixty patients with confirmed SARS-CoV-2 infection were stratified according to disease severity as Mild/Moderate (n = 8), Severe (n = 30), or Critical (n = 22), and further classified by obesity status into persons without obesity (PwoO, n = 30) and persons with obesity (PwO, n = 30). Clinical severity stratification was supported by progressive deterioration in respiratory parameters, prognostic scores, inflammatory biomarkers, and in-hospital outcomes across severity groups (**Table 1**).

**Table 1:** Sociodemographic, clinical, and laboratory characteristics of COVID-19 patients stratified by disease severity.

Variable	Mild/moderate (n = 8)	Severe (n = 30)	Critical (n = 22)	Total (N = 60)	p value
Demographics					
Age (years), mean ± SD	41.1 ± 13.4	53.2 ± 14.7	55.0 ± 9.4	52.2 ±13.3	p=0.045 *
Range (min–max)	26–68	23–80	36–71	23–80	
Sex, n (%)					
Male	2 (25.0%)	14 (46.7%)	18 (81.8%)	34(56.7%)	p=0.006 **
Female	6 (75.0%)	16 (53.3%)	4 (18.2%)	26(43.3%)	
Days from symptom onset to ER, median (range)	3.0 (2–21)	7.5 (1–22)	7.5 (3–16)	7.0 (1–22)	p=0.088
Anthropometrics and obesity status					
BMI (kg/m²), mean ± SD	28.4 ± 4.7	30.9 ± 7.3	30.5 ± 3.6	30.5 ± 5.8	p=0.605
Persons with obesity (BMI ≥30), n(%)	2 (25.0%)	17 (56.7%)	11 (50%)	30(50%)	p=0.266
Comorbidities, n (%)					
Type 2 diabetes mellitus	1 (12.5%)	9 (30.0%)	10 (45.5%)	20(33.3%)	p=0.205
Systemic arterial hypertension	0 (0.0%)	13 (43.3%)	8 (36.4%)	21(35.0%)	p=0.073
Dyslipidemia	0 (0.0%)	0 (0.0%)	7 (31.8%)	7 (11.7%)	p=0.001 **
Cardiovascular disease	0 (0.0%)	4 (13.3%)	1 (4.5%)	5 (8.3%)	p=0.346
Chronic kidney disease	1 (12.5%)	0 (0.0%)	1 (4.5%)	2 (3.3%)	p=0.200
Active smoking	1 (12.5%)	6 (20.0%)	6 (27.3%)	13(21.7%)	p=0.653
Vital signs at admission, median (range)					
Respiratory rate (breaths/min)	18.0 (14–24)	23.0 (13–34)	33.5 (22–60)	—	p<0.0001 ****
SpO_2_ (%)	94.0 (88–96)	89.0 (38–96)	66.0 (46–91)	—	p<0.0001 ****
Heart rate (bpm)	98.5 (84–120)	101.5 (69–155)	113.0 (76–138)	—	p=0.156
SBP (mmHg)	123.0 (110–147)	130.5 (84–180)	131.5 (97–164)	—	p=0.969
Temperature (°C)	36.9 (35.8–38.5)	37.2 (36.0–38.7)	36.6 (35.9–38.5)	—	p=0.270
PaO_2_/FiO_2_ ratio	304.8 (264–305)	250.5 (95–529)	122.5 (52–452)	—	p<0.0001 ****
Prognostic scores at admission, median (range)					
qSOFA	0.0 (0–1)	1.0 (0–2)	1.0 (1–2)	—	p=0.0002 ***
NEWS2	2.5 (1–5)	7.5 (3–13)	10.0 (7–13)	—	p<0.0001 ****
SOFA	0.0 (0–2)	2.0 (0–5)	4.0 (1–8)	—	p<0.0001 ****
APACHE II	2.0 (0–11)	9.5 (3–16)	12.0 (4–27)	—	p=0.0004 ***
PSI/PORT	40.0 (16–58)	66.5 (23–121)	86.0 (37–152)	—	p<0.0001 ****
MuLBSTA	1.0 (0–7)	10.0 (5–16)	7.0 (5–14)	—	p<0.0001 ****
Laboratory parameters at admission, median (range)^a^					
Leukocytes (×10³/µL)	7461 (6700–18250)	5650 (2200–18200)	9850 (5400–16400)	—	p=0.0006 ***
Lymphocytes (×10³/µL)	1488 (322–2087)	938 (370–1717)	804 (218–3230)	—	p=0.271
Neutrophil/lymphocyte ratio	3.6 (2.1–11.5)	5.8 (0.8–29.5)	7.9 (3.9–50.0)	—	p=0.006 **
Hemoglobin (g/dL)	16.4 (14.5–18.5)	14.8 (10.9–18.0)	13.2 (8.9–17.0)	—	p=0.0009 ***
Platelets (×10³/µL)	218 (185–342)	200 (100–597)	286 (33–483)	—	p=0.027 *
BUN (mg/dL)	13.1 (10.8–15.4)	13.1 (6.1–52.0)	23.0 (10.1–170.0)	—	p=0.004 **
Creatinine (mg/dL)	0.8 (0.6–1.1)	0.8 (0.0–1.7)	0.9 (0.5–23.5)	—	p=0.895
ALT (U/L)	24.4 (17.4–31.4)	35.6 (12.3–155.0)	39.0 (16.5–1077.0)	—	p=0.183
Albumin (g/dL)	4.2 (3.6–4.8)	4.0 (2.8–7.4)	3.1 (2.2–4.1)	—	p<0.0001 ****
CRP (mg/dL)	8.3 (4.3–12.3)	7.8 (0.2–28.4)	17.6 (2.8–34.5)	—	p=0.005 **
Ferritin (ng/mL)	336 (216–456)	336 (51–3863)	704 (154–14801)	—	p=0.008 **
LDH (U/L)	218 (187–248)	292 (186–647)	486 (231–745)	—	p=0.005 **
D-dimer (µg/mL FEU)	546 (420–672)	514 (190–4513)	1324 (442–125519)	—	p<0.0001 ****
Prothrombin time (s)	10.4 (10.4–10.9)	12.3 (10.3–47.3)	12.4 (9.7–15.0)	—	p=0.0008 ***
In-hospital outcomes, n (%)					
Mechanical ventilation	0 (0.0%)	4 (13.3%)	18 (81.8%)	22(36.7%)	—
Requirement for prone positioning	0 (0.0%)	3 (10.0%)	11 (50.0%)	14(23.3%)	—
In-hospital mortality	0 (0.0%)	3 (10.0%)	8 (36.4%)	11(18.3%)	—
Discharge/recovery	8 (100.0%)	27 (90.0%)	11 (50.0%)	46(76.7%)	—

Continuous variables are presented as mean ± standard deviation (SD) for normally distributed data or median (minimum–maximum range) for non-normally distributed data. Categorical variables are presented as absolute frequencies and percentages (n, %). Between-group comparisons used the Kruskal–Wallis test for continuous variables and the χ² test or Fisher’s exact test for categorical variables.

a Laboratory values available for n = 54 patients (complete blood count, n = 60; extended metabolic panel, n = 54).

—Not applicable or data not stratified to the subgroup level.

BMI, body mass index; ER, emergency room, SBP, systolic blood pressure; SpO_2_, peripheral oxygen saturation; PaO_2_/FiO_2_, arterial oxygen tension/fraction of inspired oxygen ratio; qSOFA, Quick Sequential Organ Failure Assessment; NEWS2, National Early Warning Score 2; SOFA, Sequential Organ Failure Assessment; APACHE II, Acute Physiology and Chronic Health Evaluation II; PSI/PORT, Pneumonia Severity Index/Patient Outcomes Research Team; MuLBSTA, Multivariable Prediction Model for 90-day mortality in viral pneumonia; BUN, blood urea nitrogen; ALT, alanine aminotransferase; CRP, C-reactive protein; LDH, lactate dehydrogenase.

p values: *p<0.05; **p<0.01; ***p<0.001; ****p<0.0001.

The mean age was 52.2 ± 13.3 years (range 23–80), with a significant trend toward older age with increasing severity (Mild/Moderate: 41.1 ± 13.4; Severe: 53.2 ± 14.7; Critical: 55.0 ± 9.4 years; p = 0.045). Sex distribution differed significantly across severity groups (p = 0.006): the Mild/Moderate group was predominantly female (75.0%), whereas the Critical group was predominantly male (81.8%), consistent with the known sex-differential in COVID-19 severity. Comorbidity burden did not differ significantly between severity groups for type 2 diabetes mellitus (12.5%, 30.0%, 45.5%; p = 0.205) or systemic arterial hypertension (0.0%, 43.3%, 36.4%; p = 0.073), supporting the interpretation that severity stratification captures immunological state rather than comorbidity load. Dyslipidemia was exclusively present in Critical patients (31.8% vs. 0%; p = 0.001).

All prognostic scores discriminated significantly between groups (qSOFA p = 0.0002; NEWS2, SOFA, PSI/PORT, MuLBSTA p < 0.0001; APACHE II p = 0.0004), as did respiratory parameters (respiratory rate, SpO_2_, PaO_2_/FiO_2_; all p < 0.0001), validating the clinical stratification. Inflammatory laboratory markers escalated progressively with severity: CRP (8.3, 7.8, 17.6 mg/dL; p = 0.005), ferritin (336, 336, 704 ng/mL; p = 0.008), LDH (218, 292, 486 U/L; p = 0.005), and D-dimer (546, 514, 1324 μg/mL; p < 0.0001). In-hospital mortality was 0%, 10.0%, and 36.4%, respectively; 81.8% of Critical patients required mechanical ventilation.

### 3.2 Obesity independently elevates TNF-α and resistin regardless of COVID-19 severity

**Figure 1:**
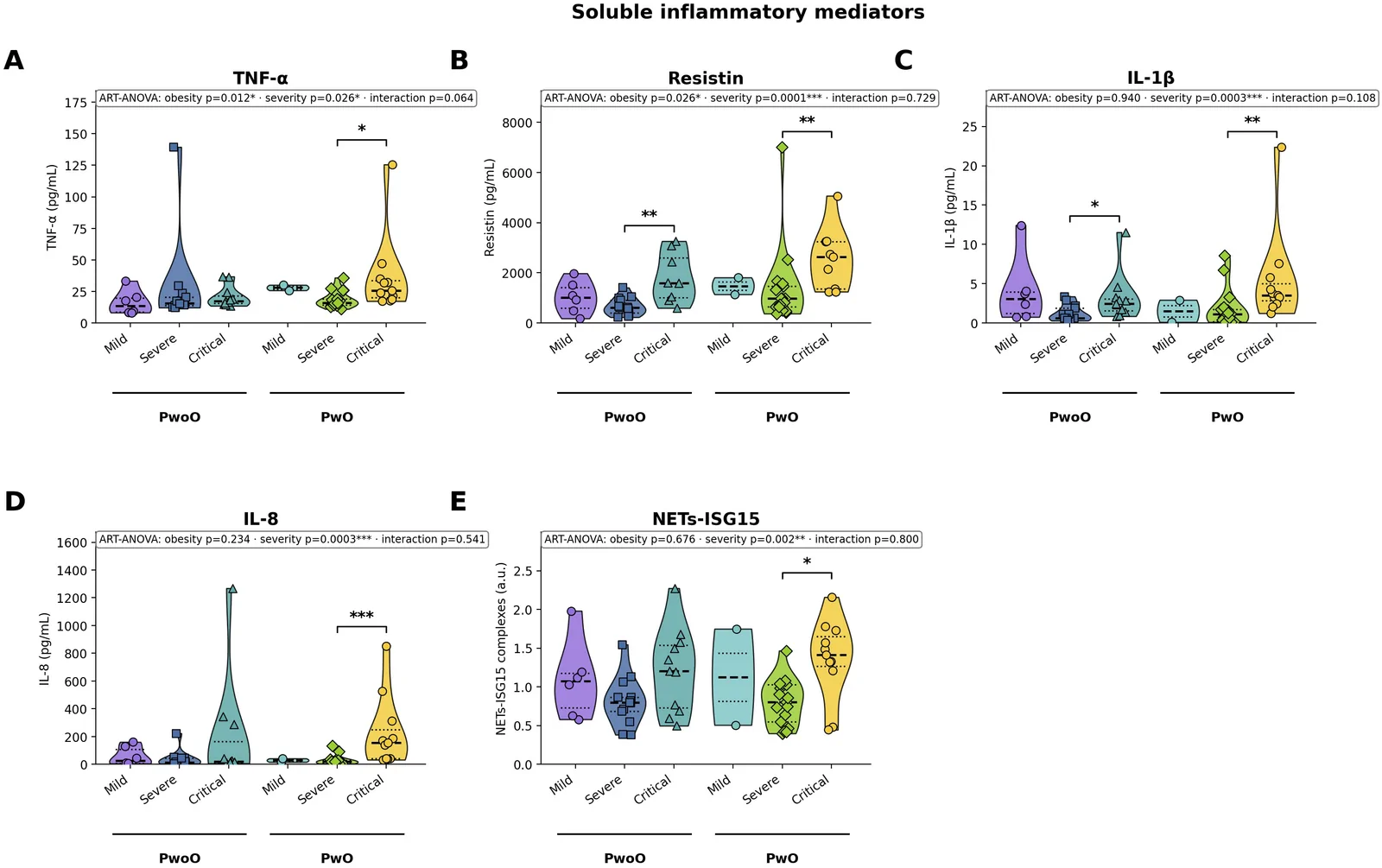
Obesity and COVID-19 severity independently shape circulating inflammatory mediators, with distinct stratum-specific amplification patterns. Violin plots show serum concentrations of **(A)** TNF-α, **(B)** resistin, **(C)** IL-1β, **(D)** IL-8, and **(E)** NETs-ISG15 complexes in COVID-19 patients stratified by obesity status (PwoO, persons without obesity, n = 30; PwO, persons with obesity, n = 30) and disease severity (Mild/Moderate, Severe, Critical). Group sizes: PwoO-Mild/Moderate n = 6, PwoO-Severe n = 13, PwoO-Critical n = 11; PwO-Mild/Moderate n = 2, PwO-Severe n = 17, PwO-Critical n = 11. Dashed lines denote the median; dotted lines denote the interquartile range; individual points are jittered for visualization. ART-ANOVA obesity, severity, and interaction p-values are shown in the upper-left inset of each panel; brackets denote significant Dunn-Holm *post-hoc* comparisons within each obesity stratum (*p < 0.05, **p < 0.01, ***p < 0.001). **(A)** TNF-α was elevated by obesity (p = 0.012) and severity (p = 0.026) as independent main effects, with a Severe-to-Critical increase confined to PwO (Dunn-Holm p = 0.016). **(B)** Resistin showed additive obesity (p = 0.026) and severity (p = 0.0001) effects, with a significant Severe-to-Critical transition in both PwoO (p = 0.008) and PwO (p = 0.009). **(C)** IL-1β was governed exclusively by severity (p = 0.0003), with a symmetric Severe-to-Critical escalation in both PwoO (p = 0.037) and PwO (p = 0.005), independent of obesity (p = 0.940). **(D)** IL-8 was severity-driven (p = 0.0003) but the Severe-to-Critical increase was restricted to PwO (p = 0.0002), with no gradient in PwoO. **(E)** NETs-ISG15 complexes followed the same pattern, severity-driven (p = 0.002) with amplification confined to PwO (p = 0.015).

Two circulating inflammatory mediators displayed obesity-associated alterations largely independent of COVID-19 severity (**Figures 1A, B**). TNF-α and resistin showed significant main effects of obesity in ART-ANOVA analyses. TNF-α levels were significantly higher in PwO relative to PwoO (obesity main effect: p = 0.012), with an independent contribution of severity (p = 0.026) and no significant interaction (p = 0.064). Within PwoO, TNF-α did not differ across severity levels (KW: p = 0.437; medians 13.62, 15.56, and 17.48 pg/mL for Mild/Moderate, Severe, and Critical, respectively). Within PwO, a significant severity gradient was observed (KW: p = 0.013), driven by the Severe-to-Critical transition: 16.15 (IQR 14.43–19.27) vs 25.87 (IQR 20.09–33.72) pg/mL (Dunn-Holm: p = 0.016). This selective severity-dependence in PwO — absent in PwoO — suggests an obesity-specific amplification of TNF-α at the critical stage.

On the other hand, resistin showed independent additive effects of both obesity (p = 0.026) and severity (p = 0.0001), with no interaction (p = 0.729). In both strata, the Severe-to-Critical transition was the decisive inflection point, within PwoO (n = 8; Dunn-Holm: p = 0.008), and within PwO (n = 9; Dunn-Holm: p = 0.009).

### 3.3 IL-1β, IL-8, and NETs-ISG15 complexes are severity-driven, with distinct stratum-specific amplification patterns

IL-1β displayed a purely severity-driven pattern (ART-ANOVA: severity p = 0.0003) with no independent effect of obesity (p = 0.940) or interaction (p = 0.108). The Severe-to-Critical transition was significant within both PwoO (KW: p = 0.017; Dunn-Holm: p = 0.037) and PwO (KW: p = 0.005; Dunn-Holm: p = 0.005), indicating that IL-1β escalation at the critical stage is a shared feature of severe COVID-19, independent of metabolic background (**Figure 1C**).

IL-8 and NETs-ISG15 also showed no significant obesity main effect (IL-8: p = 0.234; NETs-ISG15: p = 0.676) but were significantly governed by disease severity (IL-8: p = 0.0003; NETs-ISG15: p = 0.002), with no significant interaction for either variable (**Figures 1D, E**).

For IL-8, the severity effect was entirely attributable to the PwO stratum (KW: p = 0.0003), with Critical patients showing a marked elevation relative to Severe patients (Dunn-Holm: p = 0.0002). No significant severity gradient was detected within PwoO (KW: p = 0.460). For NETs-ISG15, the severity effect was likewise restricted to PwO (KW: p = 0.018): Critical patients showed higher complex abundance than Severe patients (Dunn-Holm: p = 0.015), with no significant within-stratum gradient in PwoO (KW: p = 0.180).

### 3.4 Th1 and Th17 cells undergo progressive severity-driven depletion, with Th17 additionally suppressed by obesity

**Figure 2:**
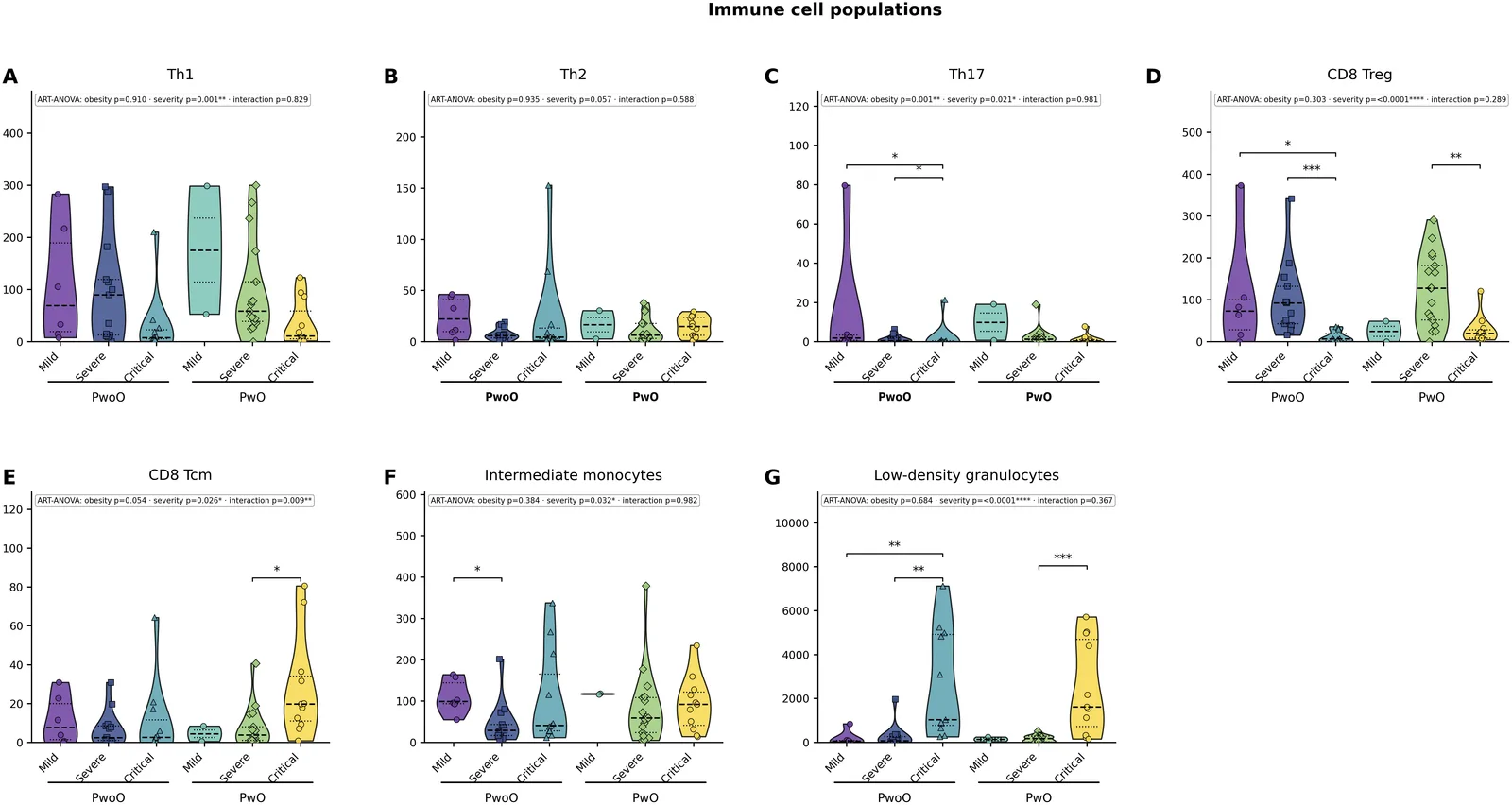
Severity-driven remodeling of circulating lymphocyte, regulatory, and myeloid populations, with obesity modulating select subsets. Violin plots show absolute counts (cells/μL) of **(A)** Th1, **(B)** Th2, **(C)** Th17, **(D)** CD8 Treg, **(E)** CD8 Tcm, **(F)** intermediate monocytes (CD14+CD16++), and **(G)** low-density granulocytes (LDGs), stratified by obesity status (PwoO, n = 30; PwO, n = 30) and disease severity (Mild/Moderate, Severe, Critical). Group sizes as in **Figure 1**. Dashed lines denote the median; dotted lines denote the interquartile range; individual points are jittered for visualization. ART-ANOVA p-values are shown in the upper-left inset of each panel; brackets denote significant Dunn-Holm *post-hoc* comparisons within each obesity stratum (*p < 0.05, **p < 0.01, ***p < 0.001). **(A)** Th1 cells declined with severity independent of obesity (severity p = 0.001; obesity p = 0.910), though no individual pairwise contrast survived adjustment. **(B)** Th2 cells were preserved across severity and obesity strata (all p > 0.05). **(C)** Th17 cells were suppressed by both obesity (p = 0.001) and severity (p = 0.021) as additive effects, with significant depletion within PwoO (Mild/Moderate vs. Critical p = 0.016; Severe vs. Critical p = 0.036). **(D)** CD8 Treg cells showed the strongest severity effect among all variables (p < 0.0001), with symmetric depletion at the critical stage in both PwoO (p = 0.0008 vs. Severe) and PwO (p = 0.003 vs. Severe), independent of obesity. **(E)** CD8 Tcm cells were the only population with a significant obesity × severity interaction (p = 0.009), showing a paradoxical expansion in critical PwO patients (p = 0.015 vs. Severe) absent in PwoO. **(F)** Intermediate monocytes declined with severity (p = 0.032), restricted to a Mild/Moderate-to-Severe drop within PwoO (p = 0.027). **(G)** LDGs showed the most pronounced severity-driven expansion of all populations (p < 0.0001), symmetric across both PwoO (p = 0.004 Critical vs. Mild/Moderate; p = 0.001 vs. Severe) and PwO (p = 0.0009 vs. Severe).

Th1 cells declined with disease severity as an independent main effect (ART-ANOVA: p = 0.001), with no contribution of obesity (p = 0.910) or interaction (p = 0.829; **Figure 2A**). The pattern was consistent across both strata. Within PwoO (KW: p = 0.041), and PwO (KW: p = 0.047). No pairwise contrasts survived the Dunn-Holm adjustment in either stratum, reflecting high within-group variability; yet the directional consistency and significant omnibus tests support a genuine severity-intrinsic Th1 contraction.

Th17 cells showed significant independent main effects of both obesity (ART-ANOVA: p = 0.001) and severity (p = 0.021), with no significant interaction (p = 0.981), indicating additive negative effects of both factors on circulating Th17 Counts (**Figure 2C**). PwoO patients maintained higher Th17 levels than PwO across severity strata. Within PwoO, a significant progressive depletion was observed (KW: p = 0.009, Dunn-Holm: Mild/Moderate vs. Critical p = 0.016; Severe vs. Critical p = 0.036). Within PwO, the same directional decline was present but did not reach significance after adjustment (KW: p = 0.109).

Th2 cells showed no significant effect of obesity (p = 0.935), severity (p = 0.057), or their interaction (p = 0.588), and no significant pairwise contrasts in either stratum (PwoO KW: p = 0.211; PwO KW: p = 0.637). Circulating Th2 cells were therefore relatively preserved across the severity spectrum. (**Figure 2B**).

### 3.5 CD8+ regulatory T cells are depleted at the critical stage

CD8 Treg cells showed the largest and most statistically robust effect among all measured populations, with a highly significant severity main effect (ART-ANOVA: p < 0.0001) and no significant obesity effect (p = 0.303) or interaction effect (p = 0.289). Within PwoO, in critical patients, these cells were significantly lower than both Severe (Dunn-Holm: p = 0.0008) and Mild/Moderate patients (p = 0.043). Within PwO, PwO-Critical had significantly fewer cells than PwO-Severe (Dunn-Holm: p = 0.003). The symmetry and magnitude of this depletion across metabolic groups argue for a severity-intrinsic mechanism independent of the adipose tissue-driven inflammatory context (**Figure 2D**).

### 3.6 CD8+ central memory T cells reveal an obesity-specific paradoxical expansion at the critical stage

CD8 Tcm cells were the only variable to exhibit a statistically significant obesity × severity interaction (ART-ANOVA: p = 0.009). Within PwoO, counts did not differ significantly across severity levels (KW: p = 0.621). In PwO, Critical patients showed a paradoxical expansion relative to Severe patients (Dunn-Holm: p = 0.015; KW: p = 0.012). This expansion (occurring against the background of global lymphopenia in critical COVID-19) may reflect aberrant reactivation of antigen-experienced memory CD8+ T cells in the context of obesity-associated chronic immune perturbation (**Figure 2E**).

### 3.7 Intermediate monocytes and low-density granulocytes respond to severe disease with distinct patterns

Intermediate monocytes (CD14+CD16++) showed a significant main effect of severity (ART-ANOVA: p = 0.032), with no significant role of obesity (p = 0.384) or interaction (p = 0.982) effects. The within-stratum difference was restricted to PwoO, where counts declined from Mild/Moderate to Severe patients (Dunn-Holm: p = 0.027), with no further significant change at the Critical stage. No significant gradient was detected within PwO (KW: p = 0.294) (**Figure 2F**).

Low-density granulocytes (LDGs) showed the most dramatic severity-associated expansion among all populations (ART-ANOVA severity main effect: p < 0.0001), with no significant obesity effect (p = 0.684) or interaction effect (p = 0.367). Within PwoO, Critical patients significantly exceeded both Mild/Moderate (Dunn-Holm: p = 0.004) and Severe patients (p = 0.001). Within PwO, PwO-Critical significantly exceeded PwO-Severe (Dunn-Holm: p = 0.0009). The symmetry of this response across obesity strata identifies LDG expansion as a critical-stage hallmark of COVID-19 pathophysiology driven by disease severity rather than metabolic background (**Figure 2G**).

## 4 Discussion

The present study demonstrates that obesity and COVID-19 severity exert largely distinct yet partially overlapping immunological effects across inflammatory, innate, and adaptive immune compartments.

These findings are contextualized within the study’s specific epidemiological setting. Patient enrolment took place between March and August 2020, corresponding to the first pandemic wave in Mexico City, during which circulating lineages were predominantly B.1 and B.1.609, with variant B.1.1.222 beginning to rise toward the end of this period (25); the lineage B.1.1.519, which subsequently dominated >80% of nationally circulating genomes and carries the spike mutations T478K, P681H, and T732A, had not yet emerged (26). The immunological patterns described here therefore reflect the host response to ancestral SARS-CoV-2 lineages, prior to the immune escape mechanisms that define later variants of concern.

This temporal specificity gains additional biological relevance when considered alongside a bioinformatic analysis of spike protein antigen presentation by Romero-López et al. (27), which identified HLA-DRB1*01:01 with a significant negative ecological correlation between the frequency of this allele and the hospitalized fatality rate across 26 Mexican states (R = −0.44, p = 0.02). The Mexican mestizo genetic background, predominant in Mexico City and shaping the local HLA allele distribution, may therefore modulate the T cell response to the SARS-CoV-2 spike in ways not fully captured by studies conducted in European or Asian populations, a dimension that warrants explicit consideration when interpreting immunophenotyping data from this cohort.

The present cohort was enrolled during the same first pandemic wave and in the same national context as two independently conducted Mexican studies that are particularly informative for interpreting our findings. Madera-Sandoval et al. characterized potential biomarkers of fatal outcome in 147 hospitalized COVID-19 patients with pre-existing comorbidities (also recruited in April–November 2020, at IMSS) and reported that hypertension and chronic renal failure — but not obesity or diabetes — were more frequent among non-survivors (28). This finding appears to challenge the assumption that obesity per se drives fatal outcomes independently of disease severity, and it aligns with our own observation that obesity status did not differ significantly across severity groups in our cohort (p = 0.266), suggesting that the immunological consequences of obesity — captured through adipokine and immune cell profiling — may be more mechanistically informative than BMI alone as a predictor of disease course. Furthermore, Madera-Sandoval et al. identified CD39 expression on T cells as a dynamic marker that increased after 7 days of treatment, reflecting T cell activation and progressive exhaustion (28); this observation converges with our findings of CD8 Treg depletion and Th17 collapse at the critical stage, both of which are consistent with a late-phase exhaustion/activation-induced apoptosis program in severe COVID-19.

One study by Torres-Ruíz et al. using the same tertiary care center setting (29) further reinforced these patterns by identifying BMI, Th1 cells, LDGs, and circulating NETs as independent predictors of severity, with other reports finding that LDGs in this population have heightened NETosis capacity with deficient DNase-mediated degradation of NET structures containing ISG-15, HMGB1, and LL-37 protein complexes (30) directly contextualizing the LDG expansion and NETs-ISG15 amplification we document at the critical stage in persons with obesity.

The Mexican population carries an exceptionally high background burden of immunometabolic risk. According to ENSANUT 2021, the prevalence of metabolic syndrome among Mexican adults reaches 45.3%, driven primarily by abdominal obesity (79.8%) and dyslipidemia — particularly hypertriglyceridemia and low HDL-cholesterol — which affects 77.1% (31). This combination creates a lipotoxic and pro-inflammatory milieu in which elevated free fatty acids and triglyceride-rich remnants activate Toll-like receptors on macrophages and monocytes, sustaining constitutive NF-κB-driven cytokine production (7). The exclusive concentration of dyslipidemia among Critical patients in our cohort (31.8% vs. 0% in Mild/Moderate or Severe; p = 0.001) is consistent with dyslipidemia acting as an active immunological amplifier that tips the balance from severe to critical multi-organ failure.

Against this backdrop, the independent elevation of TNF-α and resistin in PwO across all severity levels reflects the constitutive inflammatory state that persons with obesity bring to the encounter with SARS-CoV-2. IL-1β, in contrast, showed a purely severity-driven pattern with symmetric Severe-to-Critical escalation in both obesity strata, positioning it as a shared hallmark of transition to critical disease rather than as an obesity-conditioned mediator (9, 11).

The obesity–COVID-19 interaction can be best understood as a two-hit model: the first hit is the chronic, low-grade inflammatory state of obese adipose tissue; the second is the acute cytokine surge triggered by SARS-CoV-2. Our data indicate these processes are largely additive rather than synergistic for most variables, as evidenced by the non-significant interaction terms. The critical exception is CD8 Tcm, which showed a paradoxical obesity-specific expansion at the critical stage (interaction p = 0.009), potentially reflecting aberrant reactivation of antigen-experienced memory CD8+ T cells amid obesity-associated chronic immune perturbation. The selective amplification of IL-8 and NETs-ISG15 at the critical stage exclusively in PwO, despite the absence of a significant obesity main effect for either, suggests that the obese immune system is uniquely susceptible to pro-inflammatory amplification once systemic severity thresholds are crossed. In the context of the NETosis-LDG axis characterized by Torres-Ruíz et al. (30), the potentiated IL-8 response in critical PwO is consistent with a neutrophil-chemoattractant feed-forward loop that may drive the disproportionate thrombotic and coagulopathic risk documented in this population.

The convergence in PwO-Critical patients of IL-8 elevation (a canonical neutrophil chemoattractant), expansion of low-density granulocytes (a neutrophil population enriched for spontaneous NETosis), and increased circulating NETs-ISG15 complexes delineates a coherent mechanism whereby obesity amplifies neutrophilic NET-driven immunopathology at the critical stage of COVID-19. This neutrophilic amplification is compatible with the two-hit model outlined above, in which pre-existing obesity-associated inflammation lowers the threshold for immunothrombotic decompensation upon severe SARS-CoV-2 infection.

The symmetric and obesity-independent collapse of CD8 Treg cells at the critical stage (PwoO p = 0.0008; PwO p = 0.003) is consistent with profound Treg perturbation reported in severe COVID-19, attributed to activation-induced apoptosis, IL-6-mediated suppression of FoxP3, and redistribution to inflamed tissues (32). The near-complete collapse of circulating Th17 cells at the critical stage significant in PwoO (KW p = 0.009) likely reflects activation-induced cell death or IL-6-mediated disruption of the Th17/Treg balance (1). The constitutively lower Th17counts across all severity levels in PwO (obesity main effect p = 0.001) may impair mucosal barrier defense at early infection stages, contributing to worse outcomes in this group.

Several limitations warrant acknowledgment: the cross-sectional design, dyslipidemia as an unadjusted confounder at the critical stage, and a significant sex imbalance across severity groups (75% female in Mild/Moderate vs. 81.8% male in Critical; p= 0.006) that could not be resolved given the sample size. Additionally, the small overall cohort (N = 60) and the pronounced imbalance among some severity/obesity subgroups, particularly PwO-Mild/Moderate (n = 2) and PwoO-Mild/Moderate (n = 6), likely limited statistical power for comparisons involving the Mild/Moderate stratum; results from these subgroups should therefore be interpreted with caution pending replication in larger, more balanced cohorts.

A further limitation is the lack of systematically recorded treatment data (corticosteroids, antivirals, anticoagulation, respiratory support), which precluded assessment of their potential influence on the immunological parameters measured.

Despite these limitations, this study provides a comprehensive factorial characterization of the adipokine–immune interplay across the full spectrum of COVID-19 severity in a Mexican cohort, contributing to the growing body of evidence from Mexican institutions on the immunological determinants of COVID-19 outcomes in a population with uniquely high immunometabolic risk. Also, given the time of patient recruitment, we could not analyze the effects of vaccination in this context, which could modify cell subsets, particularly within memory cell compartments.

## Data Availability

The raw data supporting the conclusions of this article will be made available by the authors, without undue reservation.
