# Supplementary material for: Obesity and COVID-19 severity independently shape adipokine dysregulation and immune cell remodeling
Source: Front Immunol. 2026 Sep 8;17:1890251. doi: 10.3389/fimmu.2026.1890251 (PMC13593443; doi:10.3389/fimmu.2026.1890251)
Supplement: Supplementary file 1 [file Table1.docx]

| Fluorochrome-coupled antibody | Brand and catalog number |
| --- | --- |
| CD3-APC/Fire-750 | BioLegend, California, USA (Cat. 344840) |
| CD4-Alexa Fluor 488 | BioLegend, California, USA (Cat. 317420) |
| CD8-PE/Dazzle 594 | BioLegend, California, USA (Cat. 344744) |
| CD10-PE | BioLegend, California, USA (Cat. 312204) |
| CD14-PerCP | BioLegend, California, USA (Cat. 325632) |
| CD15-FITC | BioLegend, California, USA (Cat. 301904) |
| CD16-Alexa Fluor 700 | BioLegend, California, USA (Cat. 302026) |
| CD25-BV421 | BioLegend, California, USA (Cat. 302630) |
| CD45RO-FITC | BioLegend, California, USA (Cat. 304242) |
| CD62L-PE/Cy5 | BioLegend, California, USA (Cat. 304808) |
| CD127-BV650 | BioLegend, California, USA (Cat. 351326) |
| CCR7-PE/Dazzle 594 | BioLegend, California, USA (Cat. 353236) |
| IFN-γ-APC | BioLegend, California, USA (Cat. 506510) |
| IL-4-PE | BioLegend, California, USA (Cat. 500810) |
| IL-17-BV421 | BioLegend, California, USA (Cat. 512322) |
