# Supplementary material for: Obesity and COVID-19 severity independently shape adipokine dysregulation and immune cell remodeling
Source: Front Immunol. 2026 Sep 8;17:1890251. doi: 10.3389/fimmu.2026.1890251 (PMC13593443; doi:10.3389/fimmu.2026.1890251)
Supplement: Supplementary file 2 [file Table2.docx]

**Statistical analysis of immune variables in COVID-19 patients stratified by obesity and severity**

**Design:** two-factor analysis on 12 immune variables in N=60 patients (PwoO=30, PwO=30) stratified by COVID-19 severity (Mild / Severe / Critical).

**Methods:** Per-group descriptives are reported as median (Q1–Q3). Omnibus testing used the Aligned Rank Transform ANOVA (ART-ANOVA, Wobbrock et al. 2011) with factors obesity (PwoO/PwO) and severity (Mild/Severe/Critical) and their interaction. Within each obesity stratum, severity was tested with the Kruskal–Wallis test followed by Dunn’s pairwise post-hoc with Holm step-down adjustment.

| **Variable** | **PwoO–Mild** | **PwoO–Severe** | **PwoO–Critical** | **PwO–Mild** | **PwO–Severe** | **PwO–Critical** | **ART-ANOVA p**  **(obesity / severity / interaction)** | **KW within PwoO**  **(p · sig. pairs, Dunn–Holm)** | **KW within PwO**  **(p · sig. pairs, Dunn–Holm)** |
| --- | --- | --- | --- | --- | --- | --- | --- | --- | --- |
| **TNF-α (pg/mL)** | 13.62 (8.75–19.82)  n=6 | 15.56 (13.97–20.59)  n=13 | 17.48 (15.57–21.69)  n=11 | 27.98 (26.91–29.05)  n=2 | 16.15 (14.43–19.27)  n=17 | 25.87 (20.09–33.72)  n=11 | 0.012 ***** / 0.026 ***** / 0.064 | KW p=0.437  none | KW p=0.013*  S-C 0.016* |
| **Resistin (pg/mL)** | 1016.03 (593.81–1420.41)  n=6 | 611.41 (399.34–904.86)  n=13 | 1589.59 (1007.88–2598.03)  n=8 | 1475.37 (1307.43–1643.31)  n=2 | 979.81 (643.85–1470.11)  n=17 | 2625.89 (1359.01–3243.79)  n=9 | 0.026 ***** / 0.0001 ******* / 0.729 | KW p=0.011*  S-C 0.008** | KW p=0.011*  S-C 0.009** |
| **IL-1β (pg/mL)** | 3.08 (1.24–3.98)  n=6 | 0.62 (0.62–1.91)  n=13 | 2.44 (1.57–3.05)  n=11 | 1.52 (0.82–2.23)  n=2 | 1.14 (0.20–1.72)  n=17 | Excluded | 0.002 ****** / 0.117 / 0.099 | KW p=0.017*  S-C 0.037* | KW p=0.841  none |
| **IL-8 (pg/mL)** | 26.96 (8.20–107.80)  n=6 | 14.27 (9.32–45.09)  n=13 | 21.08 (13.81–164.49)  n=11 | 28.37 (21.86–34.88)  n=2 | 19.11 (11.19–27.91)  n=17 | 155.40 (42.43–249.68)  n=11 | 0.234 / 0.0003 ******* / 0.541 | KW p=0.460  none | KW p=0.0003***  S-C 0.0002*** |
| **ISG15 (pg/mL)** | 1.07 (0.73–1.18)  n=6 | 0.80 (0.68–0.87)  n=13 | 1.20 (0.73–1.54)  n=11 | 1.13 (0.82–1.44)  n=2 | 0.80 (0.55–1.03)  n=17 | 1.41 (1.27–1.65)  n=11 | 0.676 / 0.002 ****** / 0.800 | KW p=0.180  none | KW p=0.018*  S-C 0.015* |
| **Th1 (cells/µL)** | 69.47 (19.51–189.52)  n=6 | 90.23 (12.99–119.28)  n=13 | 7.90 (5.47–23.09)  n=11 | 175.86 (114.41–237.31)  n=2 | 59.02 (39.17–115.29)  n=17 | 11.01 (5.52–59.03)  n=11 | 0.910 / 0.001 ****** / 0.829 | KW p=0.041*  none | KW p=0.047*  none |
| **Th2 (cells/µL)** | 22.27 (10.01–41.08)  n=6 | 6.11 (3.73–7.49)  n=13 | 4.55 (3.04–13.27)  n=11 | 16.64 (9.72–23.57)  n=2 | 6.53 (3.25–17.78)  n=17 | 14.96 (6.19–23.71)  n=11 | 0.935 / 0.057 / 0.588 | KW p=0.211  none | KW p=0.637  none |
| **Th17 (cells/µL)** | — (excl) | 0.68 (0.23–2.04)  n=13 | 0.06 (0.01–0.41)  n=11 | 9.97 (5.38–14.56)  n=2 | 1.31 (0.88–2.38)  n=17 | 0.52 (0.17–1.02)  n=11 | n/a / 0.005 ****** / 0.577 | KW p=0.012*  S-C 0.012* | KW p=0.109  none |
| **CD8 Treg (cells/µL)** | 73.46 (28.77–100.02)  n=6 | 92.58 (42.86–132.21)  n=13 | 6.79 (2.39–19.11)  n=11 | 24.64 (12.32–36.97)  n=2 | 127.76 (52.06–182.12)  n=17 | 19.80 (8.67–28.20)  n=11 | 0.303 / <0.0001 ******** / 0.289 | KW p=0.0009***  M-C 0.043*; S-C 0.0008*** | KW p=0.002**  S-C 0.003** |
| **CD8 Tcm (cells/µL)** | 7.81 (1.50–20.02)  n=6 | 2.61 (1.06–8.48)  n=13 | 2.67 (0.00–11.66)  n=11 | 4.48 (2.51–6.46)  n=2 | 3.92 (1.03–8.08)  n=17 | 19.77 (11.03–34.11)  n=11 | 0.054 / 0.026 ***** / 0.009 ****** | KW p=0.621  none | KW p=0.012*  S-C 0.015* |
| **Intermediate monocytes (cells/µL)** | 99.34 (94.13–144.25)  n=6 | 29.45 (16.89–43.52)  n=13 | 41.16 (27.74–165.09)  n=11 | 117.52 (117.02–118.01)  n=2 | 59.28 (23.90–108.46)  n=17 | 92.66 (41.50–121.67)  n=11 | 0.384 / 0.032 ***** / 0.982 | KW p=0.027*  M-S 0.027* | KW p=0.294  none |
| **LDGs (cells/µL)** | 71.42 (31.74–98.54)  n=6 | 76.12 (33.39–266.64)  n=13 | 1039.50 (773.72–4916.50)  n=11 | 144.76 (92.15–197.38)  n=2 | 180.96 (29.76–286.00)  n=17 | 1628.40 (731.38–4694.10)  n=11 | 0.684 / <0.0001 ******** / 0.367 | KW p=0.0005***  M-C 0.004**; S-C 0.001** | KW p=0.001**  S-C 0.0009*** |

Significance code: * p<0.05, ** p<0.01, *** p<0.001, **** p<0.0001 · M = Mild, S = Severe, C = Critical
